# Supplementary material for: Adapting user-centered design principles to improve communication of peer parent narratives on pediatric tracheostomy
Source: BMC Med Inform Decis Mak. 2022 Jul 25;22:197. doi: 10.1186/s12911-022-01911-9 (PMC9316812; doi:10.1186/s12911-022-01911-9)
Supplement: Supplementary file 1 — Additional file 1: Appendix A. Standards for Reporting Qualitative Research (SRQR). Appendix B. 15 Forecasting Errors (Phase 1). Appendix C. Booklet Revision Summary (Phase 2). [file 12911_2022_1911_MOESM1_ESM.pdf]

Appendix A

Standards for Reporting Qualitative Research (SRQR)

O'Brien BC, Harris IB, Beckman TJ, Reed DA, Cook DA. Standards for reporting qualitative research: a synthesis of recommendations. Acad Med. 2014;89(9):1245–51

No.	Topic	Item
**Title and Abstract**		
S1	Title	Adapting User-Centered Design Principles to Improve Communication of Peer Parent Narratives on Pediatric Tracheostomy
S2	Abstract	**Background:** Parents who have to make tracheostomy decisions for their critically ill child may face forecasting errors and wish to learn from peer parents. We sought to develop an intervention with peer parent narratives to help parents anticipate and prepare for future challenges before making a decision.<br>**Methods:** To ensure that the intervention reflects parents' needs (rather than experts' opinions), we adapted a user-centered design (UCD) process to identify decision-critical information and refine the presentation format by interviewing parents who had tracheostomy decision making experience. Phase 1 (*n* = 10) presented 15 possible forecasting errors and asked participants to prioritize and justify the problematic ones. It also asked participants to comment on the draft narratives and preferred delivery mode and time of the intervention. Phase 2 (*n* = 9 additional parents and 1 previous parent) iteratively collected feedback over four waves of user interviews to guide revisions to the informational booklet.<br>**Results:** Phase 1 revealed that parents wanted information to address all forecasting errors as soon as tracheostomy becomes an option. They also highlighted diverse family situations and the importance of offering management strategies. The resulting prototype booklet contained five sections: introduction, child's quality of life, home care, practical challenges, and resources. Feedback from Phase 2 focused on emphasizing individualized situations, personal choice, seriousness of the decision, and caregiver health as well as presenting concrete illustrations of future challenges with acknowledgement of positive outcomes and advice. We also learned that parents preferred to use the booklet with support from the care team rather than read it alone.<br>**Conclusions:** A UCD process enabled inclusion of parental perspectives that were initially overlooked and tailoring of the intervention to meet parental expectations. Similar UCD-based approaches may be valuable in the design of other types of patient communications (e.g., decision aids).
**Introduction**		
S3	Problem formulation	With advances in medicine, tracheostomy placement has been increasingly performed in critically ill pediatric patients to enable long-term breathing support [1]. However, making a tracheostomy decision for a critically ill child is challenging for parents in various ways. Many tracheostomy decisions imply serious and

		irreversible outcomes, entailing a choice among options such as accepting a life-support-dependent future for the child, prioritizing the child's comfort and allowing death, and indefinite hospitalization [2]. Risks and benefits of each option preclude this from being a straightforward decision and each has significant long-term implications for the child and the family [2,3].<br><br>However, parents may not always receive desired information to evaluate and prepare for tracheostomy [3,4]. This makes them vulnerable to forecasting errors (i.e., inaccurate estimation of future experiences and/or emotions) [5–7], which may result in dissatisfaction with the decision process and outcomes [8]. Many parents perceive that learning the perspectives of other parents who have been in the same situation will provide informational and emotional support, but they often do not have an opportunity to talk with such peers [4,9].<br><br>Recognizing experts' limited ability to understand parents' perspectives, we turned to a user-centered design (UCD) process, in which the iterative product development is guided by end users' feedback (e.g., users' goals, needs, and interactions with the prototype) so that the final product will be effective for intended users [10–12].
S4	Purpose or research question	To correct this problem, we sought to create an intervention that used parent narratives to *inform* parents about important considerations (challenges in particular) of quality of life and typical life experiences with a child with a tracheostomy. The secondary purpose of the intervention was to *comfort* parents by normalizing emotional and practical challenges during and after tracheostomy decision making.<br><br>Our primary goal of this UCD study was to optimize the usefulness of the intervention, i.e., to create representative narratives based on real experiences that specifically address different forecasting errors. Its secondary purpose was to improve usability. In this paper, we report how we adapted these UCD principles in two distinct phases to develop our intervention to aid pediatric tracheostomy decisions with maximal parental input. Phase 1 aimed to identify important information needs to address in the intervention and obtain feedback on draft narratives. Phase 2, which consisted of four waves of 2–4 individual user interviews each, focused on eliciting feedback on the prototype intervention.
**Methods**		
S5	Qualitative approach and research paradigm	According to ISO 9241-210 (2010) as cited in [12], UCD embodies the following principles: "1) The design is based upon an explicit understanding of users, tasks and environments; 2) Users are involved throughout design and development; 3) The design is driven and refined by user-centered evaluation; 4) The process is iterative; 5) The design addresses the whole user experience; 6) The design team includes multidisciplinary skills and perspectives." User-centered design has been applied and recommended in many domains, for instance, website [13] and software development [14], new product development [15], education [16], and health care such as health information technology and tool development [17–19].

		Phase 1 aimed to identify important information needs to address in the intervention and obtain feedback on draft narratives. Phase 2, which consisted of four waves of 2–4 individual user interviews each, focused on eliciting feedback on the prototype intervention.<br><br>We leveraged our knowledge of pediatric tracheostomy decisions and parents' needs through literature review, clinical experience, and our prior research including semi-structured interviews of parents who made a tracheostomy decision [21] and a survey experiment on narratives [22] to start the design process. We sought input from parents to better understand their needs and use context of the intervention and involved them in content identification and organization of the intervention via direct contact with the materials and in-depth semi-structured user interviews. We used an iterative design process (i.e., two design phases and four iterations in Phase 2), allowing us to repeatedly incorporate parents' feedback and update the intervention for more feedback. We did not aim to develop a complete decision aid for parents to make tracheostomy decisions, but we were open-minded about what to include for informing parents about the tracheostomy option.<br><br>Given the goals of this study, the analytic process focused on critical feedback insights rather than a broader thematic analysis. Our reporting of data was focused on key takeaways rather than quotes. Phase 1 data were analyzed to develop the prototype intervention. In Phase 2, we analyzed and discussed feedback to revise the intervention after each wave of user interviews. This process continued until the team was confident about the intervention's purposes, its design, and its applicability to this population.
S6	Researcher characteristics and reflexivity	The design team included the following personnel with diverse expertise that would benefit this research: 1) decision scientists (HY, BZ-F), who have decision making expertise, research experience with parents making tracheostomy decisions (HY), and design expertise (BZ-F); 2) a neonatologist (SK) and a pediatric and palliative care physician (KP), who have rich experience working with parents, critically ill children, and tracheostomy decisions; 3) a clinical psychologist (PD), who has substantial research experience with parents who made a tracheostomy decision via previous interview research; 4) a nurse scientist (CA-E), who has extensive expertise in patient and provider decision making as well as nursing, a critical part in long-term care of patients with tracheostomy, and previously analyzed interviews of parents who made a tracheostomy decision.<br><br>The first author (HY) conducted all the user interviews. She did not have in-depth interactions with the participants prior to the user interviews, except for a few interactions with four of the participants in the previous interview study and recruitment of participants for this study. The undergraduate research assistants did not interact with the participants directly during the user interviews. They all strictly followed the interview guide and note-taking protocol to minimize any potential bias.

S7	Context	We conducted 20 semi-structured user interviews (*n* = 10 for each design phase) between September 2019 and February 2020 at convenient mutually agreed-upon locations by the parents and interviewer.
S8	Sampling strategy	To recruit parents who were experienced with preference-sensitive tracheostomy decisions and could offer diverse perspectives, we sought to recruit parents who met the following eligibility criteria: 1) were fluent in English and were at least 18 years of age at the time of enrollment; 2) had made a tracheostomy decision for their child, with or without chronic mechanical ventilation, between about six months and three years prior to the interview; and 3) considered themselves knowledgeable about the child's condition and services, both at the time of the tracheostomy decision and afterwards. The child whose tracheostomy decision had been made should have met the following criteria: 1) was less than 18 years of age at the time of decision making; 2) had a life-limiting illness, in which either a decision to pursue or a decision not to pursue tracheostomy would be ethically appropriate; and 3) was a current patient or had been a patient at the time of tracheostomy decision making at a large Midwestern academic children's hospital and regional referral center for tracheostomy and home ventilation. The current state of the child could be one of the following: 1) if a tracheostomy was pursued: alive, subsequently decannulated, or deceased (more than six months ago prior to the interview, in order not to interrupt the bereavement process); 2) if a tracheostomy was declined at that time of decision making: alive or deceased (more than six months ago prior to the interview).<br><br>Eligible parents were recruited by one of the following ways: 1) clinicians' recommendations of parents who would potentially meet the above criteria by medical chart review; 2) a mass email to a group of parent volunteers sent by a volunteer coordinator at the study site; 3) flyers and brochures distributed on the study site. For potentially eligible and interested parents, their children's relevant medical information and their responses to the screening questions were reviewed before enrollment to ensure their eligibility and suitability for the study.<br><br>We conducted 20 semi-structured user interviews (*n* = 10 for each design phase) between September 2019 and February 2020 at convenient mutually agreed-upon locations by the parents and interviewer…Each participant was compensated $50 for the study plus travel incentives.
S9	Ethical issues pertaining to human subjects	The study was declared exempt research by the University of Michigan Medical School Institutional Review Board (IRBMED).
S10	Data collection methods	Phase 1 aimed to identify important information needs to address in the intervention and obtain feedback on draft narratives. Phase 2, which consisted of four waves of 2–4 individual user interviews each, focused on eliciting feedback on the prototype intervention.<br><br>We used an iterative design process (i.e., two design phases and four iterations in Phase 2), allowing us to repeatedly incorporate parents' feedback and update the intervention for more feedback.

		We conducted 20 semi-structured user interviews (*n* = 10 for each design phase) between September 2019 and February 2020 at convenient mutually agreed-upon locations by the parents and interviewer. In addition to the interviewer, an undergraduate research assistant was present to take notes to facilitate data analysis…The first author (HY) conducted all the user interviews. She did not have in-depth interactions with the participants prior to the user interviews, except for a few interactions with four of the participants in the previous interview study and recruitment of participants for this study. The undergraduate research assistants did not interact with the participants directly during the user interviews. They all strictly followed the interview guide and note-taking protocol to minimize any potential bias.<br><br>**Phase 1 Procedure:**<br>We asked parents for their consent to participate in the study and started the interview. At the end, parents filled out a demographics and experiences survey.<br><br>**Phase 2 Procedure:**<br>Similar to Phase 1, after obtaining informed consent, we started the interview. (Step 1 was omitted for the parent who had participated in Phase 1.) At the conclusion of the interview, parents completed a survey of demographics and tracheostomy-related experiences.
S11	Data collection instruments and technologies	**Phase 1 Materials:**<br>Based on our review of the literature, clinical experience, and previous research [21,22], we developed 15 single-sentence descriptions of forecasting errors in four key domains where forecasting errors might be common and more information support is warranted: quality of life, home care skills, material constraints, and impact on family life (Additional file 1: Appendix B). These descriptions represented candidates of problems for participants to help us identify important issues to address.<br><br>To begin with, parents reported valuing quality of life when making medical decisions for their children [23], but they may encounter *focusing illusion* (i.e., overemphasizing certain considerations and overlooking others) [24] and *misconstrual* of what the future may entail (as discussed in [25]) when evaluating the child's quality of life. We constructed five descriptions to illustrate potential biases, such as focusing on survival rather than other consequences for critically ill children [2,26], anticipating regret and guilt if they had not tried everything for survival [27], and being overly optimistic about the long term [9,28,29].<br><br>In addition, parents assume a natural caregiving duty for their child and underestimate challenges of their child's complex home care needs [29]. Parents do not feel fully prepared for transitioning the care to home, handling emergencies and complications, and coordinating health care visits [4,30]. Accordingly, we constructed three items to illustrate underestimation of the burden of home care commitment.

		Professional and social support is important to sustain the care for a child with tracheostomy needs. However, resources may not be as easily accessible as parents expect, for example, a lack of suitable housing [31] and support from the community [9]. Parents may also overestimate the quantity and quality of home nursing care, as they feel stressed about inadequate nursing coverage to maintain their own well-being [30,32,33]. We constructed four items to describe these forecasting errors.<br><br>Furthermore, caring for a child with a tracheostomy has impact on the family's income, relationships, and social life. Some families experience employment and financial struggles [30,32,34]. Strained family relationships (i.e., marriage, parent-child) and social isolation were found associated with caregiving responsibilities of a child with a tracheostomy [9,31,35]. We created three items to describe these issues.<br><br>For each of the 15 forecasting errors, we constructed a short narrative. According to the Narrative Immersion Model [36], a constructed narrative can be as effective as a real story as long as it appears realistic, credible, and engaging. Experts including decision scientists (HY, BZ-F) and clinicians who are involved in pediatric tracheostomy decisions (SK, KP) reviewed the narratives to ensure that they captured the core ideas of the corresponding forecasting errors and presented accurate medical knowledge and realistic patient experiences without medical jargon.<br><br>The interview guide included four steps. Step 1 asked parents about their tracheostomy decision experience to prepare them for the study. It contained questions that asked about any difficulties during the decision process and their information needs. The tracheostomy decision process can be stressful and overwhelming to talk about. Therefore, we designed these questions to not only understand the decision making environment (the context in which the intervention will be used), but also guide parents to think about their challenges when making the decision.<br><br>Steps 2 and 3 primarily focused on improving the usefulness of the future intervention. Step 2 was a card sorting task in which parents were asked to think aloud and comment on how problematic these 15 forecasting errors, if occurred, would be for parents to make informed decisions. We asked them to sort the forecasting errors into three categories: very problematic, somewhat problematic, and not problematic. After card sorting, parents were given opportunities to revise their sorting and comment more on each category of the cards. We further asked them if these issues were realistic and if we missed any problematic forecasting errors. While all of these forecasting errors are possibly relevant to tracheostomy decisions, our goal was to identify which errors were viewed by parents as the most critical to address. This step sought to understand parents' experiences and involve them in the identification of suitable content for the intervention.

		In Step 3, we showed parents narratives that corresponded to the forecasting errors they perceived to be the most problematic and asked them whether they thought the narratives conveyed the core issues. We also elicited actionable feedback about what they would keep or change in the narratives. This step was designed to include parents in developing narratives for the prototype intervention.<br><br>In Step 4, we sought feedback about how and when such information should be delivered. This would help us set the tone and format of the prototype intervention, further understand the task environment, and increase usability.<br><br>**Phase 2 Materials:**<br>The prototype booklet of narratives (Table 3) consisted of five sections. To make it easy for parents to understand and follow, each section was organized by key takeaway points with narratives in simple vocabulary and large prints. The initial concept is that parents could read the booklet before a meeting and take home as appropriate. The goal of Phase 2 was to revise the booklet iteratively based on parents' and professionals' feedback.<br><br>Step 1 was the same as that in Phase 1. Step 2 aimed to obtain parents' perceptions of important, concerning, and unnecessary parts of the booklet so that we would know what to keep, change, or add. These questions were designed to improve the usefulness of the booklet, acquiring an explicit evaluation and understanding of whether the booklet adequately conveyed parents' experiences and held value for helping future parents understand these topics. We also asked parents about their perception of the length of the booklet and welcomed them to edit the booklet to increase usability as well. In the second and third waves, we focused more on probing less important points that could be removed and the third wave added one question about whether to take out or combine stories in the "support from schools and public" section. Step 3, which asked more specifically about the booklet, was similar to Step 4 in Phase 1.
S12	Units of study	We conducted 20 semi-structured user interviews (*n* = 10 for each design phase) between September 2019 and February 2020 at convenient mutually agreed-upon locations by the parents and interviewer.<br><br>Ten parents were interviewed for Phase 1. Parent and child characteristics are reported in Tables 1 and 2.<br><br>Parent and child characteristics for Phase 2 are reported in Tables 1 and 2.
S13	Data processing	The audio recordings were transcribed verbatim, de-identified, and checked by two research assistants for completeness and accuracy. The first author (HY) organized interview notes after each user interview.
S14	Data analysis	Given the goals of this study, the analytic process focused on critical feedback insights rather than a broader thematic analysis. Our reporting of data was focused on key takeaways rather than quotes. Phase 1 data were analyzed to develop the prototype intervention. In Phase 2, we analyzed and discussed feedback to revise the

		intervention after each wave of user interviews. This process continued until the team was confident about the intervention's purposes, its design, and its applicability to this population.<br><br>**Phase 1 Data Analysis:**<br>For Steps 1 and 4, the first author (HY) organized notes of every participant's response to each question. For Step 2, she reviewed the sorting and comments for each forecasting error. For Step 3, she summarized each participant's main comments and feedback on wording for each narrative and edited the narratives. Taking all the analysis, she made a summary of important things to focus on and prepared narratives for developing the prototype. She discussed the findings and made the initial versions of the prototype booklet with the senior author (BZ-F) through a series of meetings and email exchanges. A summary of key findings and the prototype booklet were emailed to the design team members, who made wording edits and offered suggestions over email until the prototype was deemed ready for Phase 2.<br><br>**Phase 2 Data Analysis:**<br>The first author (HY) noted important feedback on the prototype booklet, made edits, and presented them to the senior author (BZ-F, via meetings or emails) and the design team (via emails) for further revision after each wave until a new version was ready for the next wave of user interviews.
S15	Techniques to enhance trustworthiness	The audio recordings were transcribed verbatim, de-identified, and checked by two research assistants for completeness and accuracy.<br><br>**Phase 1 Data Analysis:**<br>She discussed the findings and made the initial versions of the prototype booklet with the senior author (BZ-F) through a series of meetings and email exchanges. A summary of key findings and the prototype booklet were emailed to the design team members, who made wording edits and offered suggestions over email until the prototype was deemed ready for Phase 2.<br><br>**Phase 2 Data Analysis:**<br>The first author (HY) noted important feedback on the prototype booklet, made edits, and presented them to the senior author (BZ-F, via meetings or emails) and the design team (via emails) for further revision after each wave until a new version was ready for the next wave of user interviews.
**Results/Findings**		
S16	Synthesis and interpretation	**Phase 1 Results:**<br>Overall, parents reflected that all 15 forecasting errors were realistic issues that might be problematic for making an informed tracheostomy decision. Based on their feedback, we identified the following key components that should be reflected in the content and tone of the prototype intervention:

First, parents acknowledged that they should consider factors other than survival but emphasized that the decision should be made based on the child's best interest rather than convenience and needs of the parent caregivers. They reported diverse expectations and perceptions of a child's best interests. Some valued tracheostomy as a means for their child to thrive while others considered tracheostomy as a threat to their child's comfort and current active life. Either way, parents wished to know both positive and negative outcomes at the time of decision making.

Second, parents considered insufficient preparation regarding home care very problematic. Parents reported that the caregivers ought to be prepared for routine tasks. Otherwise, the caregivers would be stressed and the child's life may be at great risk due to inadequate care. Parents also reported the lack of competent and reliable nurses to provide care.

Third, parents reminded us that some forecasting errors might be more consequential for some patients' families than for others. For instance, having a child with a tracheostomy might significantly impact household income if both parents used to work, but might have little or no influence, if one parent already stayed at home. Family relationships and social life could also depend on how strong the relationships were.

Fourth, parents described that both making the decision and taking care of a child with tracheostomy could be scary and emotional. They said it would be important to get emotional support and know that other parents have similar feelings. They would not only want to know the challenges, but also support and solutions for those issues to preserve some positivity in life.

Fifth, most parents preferred to get information about tracheostomy and its influences on everyday life as soon as it became an option. Parents appreciated ample time to digest the information before doctors let them know it is necessary to make a decision. Having information prior to the family meeting would help them ask the right questions, whereas having information after the consultation would enable parents to ponder over their most relevant issues. They preferred multiple ways to deliver the information, such as a short video, pamphlet, website, and app.

Phase 2 Results:
Below, we summarize the iterative revision process by wave (See Additional file 1: Appendix C for the details of changes). Overall, parents found the booklet easy to follow and at the right length despite it being 15 pages. They considered this booklet useful to have as soon as tracheostomy becomes an option.

		Wave 1 (n = 2)<br>**Positive Feedback.** Both participant parents found the booklet easy to understand, realistic, and useful. They valued informing parents about the downsides and risks of tracheostomy because the challenges of tracheostomy should not be underemphasized and it would be important to be prepared for life changes. They appreciated diverse perspectives of child's quality of life and the information about the uncertainty of the length of tracheostomy. Both of them found the sections about home care and other practical matters useful, because they addressed realistic challenges and caregivers' own needs, which were often neglected.<br><br>**Constructive Feedback.** Both participant parents expressed the concern that the introduction made tracheostomy sound elective and recommended that a tracheostomy should be presented as essential for survival at the beginning of the booklet. Therefore, we emphasized that tracheostomy provides "a stable airway" and without it "some patients may not live long" in the first paragraph of the introduction. One parent recommended reiterating that the best choice is at the individual family's discretion after the quality-of-life narratives, because parents might doubt themselves after reading perspectives that conflicted with their values. Given all the challenges in finding support, these parents recommended telling parents who would face a tracheostomy decision that this decision would change caregivers' life and that they should be comfortable taking care of the child first before relying on other resources to support the care. They provided suggestions to make the narratives more concrete and vivid, so we added those details.<br><br>***Wave 2 (n = 2)***<br>**Positive Feedback.** Both participant parents found all the content useful. They considered the introduction helpful because it laid out technical pros and cons, main tasks, and important questions parents should think about during decision making. Both of them acknowledged the importance of diverse perspectives and individual choice. They considered the home care challenges realistic and liked the idea that parents should accept the care responsibilities first.<br><br>**Constructive Feedback.** Regarding the "life-and-death" nature of tracheostomy, one parent found our previous revision unsatisfactory, as we made "death" without a tracheostomy sound probabilistic. Thus, we used "last resort to optimize the chances for long-term survival" to illustrate the importance of this decision. One parent recommended emphasizing that the decision could be "life-altering" rather than just "you may not be able to work" as what we added in the previous wave.<br><br>Another salient issue was the balance of positive and negative narratives. While one parent had more negative perceptions of getting support and suggested adding more details about financial difficulties and family relationship changes, the other parent in this wave perceived these narratives too negative and suggested adding positive stories about home health care nurses and family's support. As our booklet focused

		on describing challenges, we added positive scenarios in the narration but not as an individual story. Both parents, however, provided some advice to deal with these challenges.<br><br>One parent suggested that parents should have a team (palliative care in particular to discuss goals of care) with them to go through the booklet rather than read it alone. This was an important suggestion about how this booklet should be used, so we added this message at the end of the introduction.<br><br>***Wave 3 (n = 2)***<br>**Positive Feedback.** Again, the participant parents applauded the descriptions of upsides and downsides of a tracheostomy and parents' thought process. The messages that every child is different and that finding a team to go through the process together were well received. Both parents considered home care and other practical challenges realistic.<br><br>**Constructive Feedback.** The balance of positive and negative aspects was still an issue. In particular, one parent had quite positive experiences. They appreciated different perspectives, but disliked the focus on negative outlook of quality of life, as some children with tracheostomies could enjoy many aspects of life. Similar to the previous wave, while these parents described more challenges, they suggested adding some advice and illustrating the adaptation for balance.<br><br>In addition to revising based on parents' feedback, we focused on generalizing key ideas in the narratives. We removed various detailed statements that parents had controversies about and details about the specific hospital they attended.<br><br>***Wave 4 (n = 4)***<br>**Positive Feedback.** The participant parents noted that the narratives not only captured peer parents' voices and tone, but were also to the point. The narrations summarized key points well. They valued different perspectives of quality of life and individual choice.<br><br>**Constructive Feedback.** As for the issue of balance, one parent suggested adding the idea that nurses could be good partners in care and some advice such as home schooling and couples counseling. Another parent suggested taking out the concrete details of home health care nurses' behaviors and including some hope (e.g., "you will have good support"). Although we could not guarantee a good outcome, we illustrated the idea of adaptation in the revision.

		Our booklet was designed to give parents information about tracheostomy early in the decision process. One parent shared her experience and pointed that later tracheostomy might not be an option anymore. We thought it would be worth presenting the idea that tracheostomy might not always be an option.
S17	Links to empirical data	Given the goals of this study, the analytic process focused on critical feedback insights rather than a broader thematic analysis. Our reporting of data was focused on key takeaways rather than quotes.<br><br>Additional file 1: Appendix C
**Discussion**		
S18	Integration with prior work, implications, transferability, and contribution(s) to the field	**Phase 1 Discussion:**<br>We decided to make a short booklet because it is a simple and efficient way to present information and it could be adapted to other formats relatively easily. Our findings shaped the content and design of the prototype booklet. First, parents wanted to make the decision for the child's best interest, not theirs, which is consistent with one of the good-parent beliefs—"putting the child's needs above my own" [23]. While the caregivers' physical and mental health is important, we realized that the booklet needed to reflect a delicate balance of describing different types of challenges and not frame the situation as a tradeoff between the interests of the child and parent caregivers. Second, since parents worried most about not knowing specific details of tracheostomy home care, we decided to provide more detailed narratives on this topic to fulfill the identified needs. Third, there appeared to exist an issue of balance of positive and negative information. As the impact of forecasting errors may depend on the family's situations, we decided to acknowledge and describe different levels of impact explicitly in the narratives. Moreover, while a focus on negative aspects of lived experiences validated parents' feelings, it could be overwhelming to learn about all the challenges without knowing ways to deal with them. Therefore, we decided to present not only the challenges, but also useful advice shared by parents. Fourth, we learned that parents wanted information as early as possible, so we strove to frame the information using an open-minded tone and to focus on getting parents think about these issues. These takeaways addressed user experience (usefulness) by attending to parents' urgent needs for diverse perspectives and solutions.<br><br>**Phase 2 Discussion:**<br>The UCD process deepened our understanding of parents' needs for messages and information and led to numerous specific changes in the tone and content to better align with parents' experiences. From Phase 1, we learned that families could make different choices depending on their values and judgments of their child's conditions and used this tone in the prototype booklet. However, parents in Phase 2 pointed out that this message should be conveyed even more strongly than our original design did. For instance, parents recommended reiterating this point at the end of the "best interest" section and reflecting more details about different kinds of positive and negative experiences in the narratives. Moreover, while we tried to avoid presenting a tradeoff between the child's and family caregivers' interests, parents in Phase 2 helped us find a

way to state the importance of caregivers' physical and mental health because the patients would suffer if the caregivers were too stressed to manage the care. Furthermore, emphasizing diverse individual situations and presenting the tracheostomy option in a nonforceful way came at the cost of downplaying serious outcomes of this decision: life and death for the child, and life alteration for the caregivers. Parents pointed out this downside and provided a solution that we could emphasize these points in certain narrations.

In addition, by interviewing parents with different experiences and backgrounds, we were able to include diverse perspectives and add details to the booklet. Parents' emphasis on concrete details forced us to make intentional choices about when to be specific and when it would be better to generalize details to make the narratives more broadly representative of key ideas. Including a variety of enriched narratives helped with the balance issue. While our goal was to focus on conveying challenges and making parents not feel alone, acknowledging positive outcomes and providing advice in the meantime appeared to be helpful for parents to digest information and feel supported.

In terms of usability, parents confirmed that the booklet was easy to understand and efficient to use. One point that emerged in this process was that although parents wanted a lot of information, it could be overwhelming to read the booklet alone without other support. Therefore, we explicitly designed the booklet as informing future conversations with providers rather than as a standalone information resource.

General Discussion:

Although the UCD process required both time and a willingness to repeatedly modify drafts in response to feedback, it enabled us to gain a direct understanding of how parents perceived forecasting errors in tracheostomy decision making and how they viewed the tone and content of the booklet. We incorporated constructive feedback based on our understanding of the literature and other parents' experiences in multiple rounds of revision. Parents regarded the intervention easy to follow and at the right length.

As we theorized that many forecasting errors were due to incomplete understanding of the challenges, most narratives described negative consequences. Parents offered firsthand information regarding quality of life and their difficulties with emergencies, home health care nurses, the community, and other family members. These examples increased concreteness of the narratives and, therefore, may help parents facing these decisions accurately envision everyday life with a child with a tracheostomy.

It was also useful to obtain parents' feedback about emphasizing the uniqueness of each case, the importance of adaptation strategies, and the need for acceptance. For instance, research shows diverse outcomes (e.g., discharge, death, decannulation, differences in developmental progress) in tracheostomy- and ventilator-dependent children [37–39]. Parents would become experts of their own children [40,41], although not all

have increased confidence and acceptance of their child's underlying condition and caring needs over time [35].

Another key goal of our booklet was to make parents not feel alone in the process by presenting challenges and strategies. Participant parents suggested that it would be crucial to maintain caregivers' well-being and establish trusting relationships with other professional parties such as home health care nurses and medical supply companies [35]. Well-being is often poor in parent caregivers of children with tracheostomies [30,42,43] and it is unrealistic that parents care for the child without any help. Seeking emotional support, resources, and information from professionals may share parents' care burden and increase confidence [40].

The design and development of this booklet successfully captured several key steps of UCD. Our approach scored 7 on the UCD-11 (a descriptive measure of user-centeredness of the design and development of personal health tools) [44] and also had a few additional elements in the DEVELOPTOOLS Reporting Checklist [45]. Our method (e.g., as illustrated by the numbers of development steps and iteration that involved users) was comparable to those of the studies included in a systematic review of UCD in patient decision aids and health tools [46]. Bearing the purposes and use context of the intervention in mind, we sought to involve parents in most stages of the iterative development of the intervention, from understanding their needs to refining the intervention.

Our approach may be applicable to other health care contexts where researchers aim to help patients and caregivers understand experiences of treatment options and considerations during decision making. Although the use of UCD is recommended for enhancing user experiences, many studies do not involve stakeholders (e.g., patients, clinicians) in developing health decision aids and technologies [47,48]. Engaging stakeholders in research can be time consuming. However, when there is a lack of understanding of stakeholders' needs and goals, it is worthwhile considering involving them in the process of design and development of patient education to ensure usefulness and usability of the materials.

Our user-centered process yielded a parent-tested tracheostomy information booklet that is ready for implementation in clinical settings. The final version is available for free download (https://deepblue.lib.umich.edu/handle/2027.42/154713). Nonetheless, further work is needed to evaluate the impact of using the booklet on improving informed decision making and decision satisfaction. We are seeking to incorporate the booklet into goals-of-care consultations for critically ill children. We will first ask clinicians who are doing these consultations to review the booklet and to discuss feasible ways to use it. As the booklet was designed to be used as early in the decision process as possible, clinicians may consider asking parents whether they would be willing to receive such information prior to family conferences to get prepared for the consultation. Clinicians may also discuss key points in the booklet during the consultation or

		provide it to parents after the consultation as an additional source of information. We encourage both providers and parents who are facing discussions of such difficult tracheostomy decisions to use this booklet when appropriate. We also appreciate feedback from providers and parents who have used it.
S19	Limitations	Given our focus on the usefulness of the intervention and parents' input, we have yet to observe how parents actually use the booklet. While we did not seek feedback on the booklet from health professionals outside our team, our team did include a few health professionals who actively counsel parents about this decision in their clinical work. In the future, we can involve an advisory panel of more stakeholders, facilitate perspective sharing between clinicians and parents, and translate the booklet into other languages.<br><br>Our study had several limitations that may affect its generalizability. Participants were limited to English-speaking parents from a single study site. Although we strove for recruiting parents from diverse backgrounds and interviewed parents with different education and income levels, most of them were Caucasian and likely had access to the substantial resources needed to manage a child with complex medical needs. In addition, they offered their own experiences with home care and life in a Midwestern state, but different states and regions may have different regulations, resources, and cultures. The generalizability of the findings and the booklet in diverse populations and different states needs to be further tested. Moreover, patients for whom a tracheostomy could be considered have diverse underlying conditions, yielding a full spectrum of prognosis. Our study was limited to more serious cases in which the children were anticipated to have motor and cognitive impairment. Other studies need to be done to investigate the information needs of parents who make tracheostomy decisions for children who are expected to be decannulated and fully recover. Nevertheless, these parents enriched our understanding of their experiences and needs and substantially improved the prototype intervention.
**Other**		
S20	Conflicts of interest	The authors declare that they have no competing interests.
S21	Funding	The study was funded by the Rackham Graduate Student Research Grant, University of Michigan. The funding source had no role in designing the study, interpreting the data, writing, or publishing the report.

Appendix B

15 Forecasting Errors (Phase 1)

Considerations of Quality of Life

1. Some parents only consider survival and fail to consider other important things.
2. Some parents focus on survival and short-term outcomes, such as benefits and risks of the tracheostomy placement procedure, but fail to consider its long-term implications, e.g., responsibilities of accepting a child who may not function independently.
3. Some parents focus on avoiding the pain of their child passing away soon but underestimate the pain of watching their child grow up without doing much (e.g., cannot speak).
4. Some parents assume having a tracheostomy will mean better long-term quality of life even though that is not always true.
5. Some parents feel that there is always hope of full recovery even though full recovery is sometimes not possible.

Home Care Skills

1. Some parents underestimate the complexity of taking care of a child with tracheostomy.
2. Some parents underestimate the amount and scariness of emergencies when taking care of a child with tracheostomy.
3. Some parents underestimate how long their child will be on tracheostomy and later have a feeling of exhaustion and endlessness.

Material Constraints

1. Some parents fail to consider whether the current home environment is suitable for a child with tracheostomy.
2. Some parents overestimate care support from the community, e.g., public facilities, schools.
3. Some parents overestimate access to and quality of home nurses.
4. Some parents fail to consider uneasiness and troubles of having a stranger (nurse) living in the same house.

Impact on Family Life

1. Some parents underestimate the impact of household income reduction.
2. Some parents underestimate the influence of having a child with tracheostomy on partner and other children in the family.
3. Some parents underestimate the influence of having a child with tracheostomy on their social life.

Appendix C

Booklet Revision Summary (Phase 2)

	Positive Feedback	Constructive Feedback	Changes
Wave 1 Parent Feedback	• Easy to understand<br>• Helpful to know the downsides and risks of tracheostomy<br>• Useful to read about diverse perspectives of quality of life and realistic estimates<br>• Realistic practical challenges<br>• Emphasis on caregivers' needs<br>• Helpful support group information readily available to parents, if used with caution	• Need to emphasize the seriousness of the tracheostomy decision<br>• Explain "technology-dependence," whether it is with or without a ventilator<br>• Reiterate that the decision could be different for different families (the "best interest" section)<br>• Emphasize that the life of parents will change<br>• Emphasize parents' confidence in home care before relying on other resources<br>• Be clear that some poor outcomes are due to the underlying disease, not tracheostomy<br>• Add details such as taking notes during tracheostomy care training, SSI, utility bills, lack of emotional support by family, and perspectives of single parents (income, family support)<br>• Take out "all hospital nurses are nice"<br>• One parent suggested taking out the description of how other people look down on your child, but the other did not.	Added major points in the narrations and details in the narratives
Wave 1 Team Feedback	• Since there were lots of added details, probe about what can be taken out (especially looks from others) in the next wave.		

Wave 2 Parent Feedback	• Good to lay out pros and cons of tracheostomy and questions parents wonder about during decision making<br>• Diverse perspectives of values<br>• Emphasis on individual choice<br>• Realistic information and summary about challenges<br>• Helpful to have support group information	• Insufficient emphasis on the seriousness of the tracheostomy decision<br>• Emphasize it is 24/7 care<br>• Tell parents to read this booklet with professionals' help<br>• Emphasize the role of palliative doctors for discussing goals of care<br>• Emphasize that tracheostomy alters caregivers' lives<br>• Emphasize difficulties in finding financial and other resources<br>• Add positive aspects of home health care nurses and help from the community and family members<br>• Add advice about involving other children in the care plan, finding ways to go out, and consulting with education intervention programs<br>• Add one support group	Added these points in appropriate places
Wave 2 Team Feedback	• Keep probing whether to combine the support from school and community narratives.		
Wave 3 Parent Feedback	• Good summary of different aspects of tracheostomy and questions to consider<br>• Good to emphasize individual choice and team support<br>• Inform parents about many important details and advice	• More descriptions about insurance types, such as private companies and Medicaid<br>• Provide more positive outlook and advice about how to select and work with suitable home health care nurses and education intervention programs<br>• Emphasize that confidence in and comfort with home care will increase over time<br>• Add details about taking work off, 2nd caregiver	Added specific points and then generalized as key ideas

		not as helpful, home health care nurses' behaviors, medical supply companies, social isolation, and challenges of going out (cumbersomeness, winter germs)<br>• Add social workers as a resource to find support groups	
Wave 3 Team Feedback	• Since details can be controversial, some details of the narratives have been generalized.<br>• Add descriptions of responsible nurses in the narratives.		
Wave 4 Parent Feedback	• Easy to read<br>• Descriptive, detailed, but at appropriate length<br>• Diverse and fine balanced perspectives<br>• All information is important and real.<br>• Powerful narratives<br>• Emphasis on individualized situations<br>• Reading these stories makes parents not feel alone.<br>• Helpful to provide support group information	• Some wording suggestions<br>• Add the idea that tracheostomy may not be an option anymore as time progresses<br>• Add the idea about training may delay going home<br>• Could be a little more balanced by telling parents that "good nurses could be partners of your child's care."<br>• Add points about transportation and couples counseling (advice)<br>• Add two support groups	Changed wording and added points accordingly
Wave 4 Team Feedback	None		
